# Supplementary material for: Genome-wide analyses of variance in blood cell phenotypes provide new insights into complex trait biology and prediction
Source: Nat Commun. 2025 May 7;16:4260. doi: 10.1038/s41467-025-59525-4 (PMC12059119; doi:10.1038/s41467-025-59525-4)
Supplement: Supplementary file 1 — Supplementary Information [file 41467_2025_59525_MOESM1_ESM.pdf]

Genome-wide analyses of variance in blood cell phenotypes provide new insights into complex trait biology and prediction

Ruidong Xiang[1-5,*], Chief Ben-Eghan[2,6-9], Yang Liu[1,2,6-9], David Roberts[10,11], Scott Ritchie[1,2,6-9], Samuel A. Lambert[1,2,6-9], Yu Xu[2,6-9], Fumihiko Takeuchi[1,12] and Michael Inouye[1,2,6-9,*]

1. Cambridge Baker Systems Genomics Initiative, Baker Heart and Diabetes Institute, Melbourne, Victoria, Australia
2. Cambridge Baker Systems Genomics Initiative, Department of Public Health and Primary Care, University of Cambridge, Cambridge, UK
3. Agriculture Victoria, AgriBio, Centre for AgriBioscience, Bundoora, Victoria 3083, Australia
4. The School of Applied Systems Biology, La Trobe University, Melbourne, VIC, 3086, Australia
5. Baker Department of Cardiometabolic Health, The University of Melbourne, VIC, 3010, Australia
6. British Heart Foundation Cardiovascular Epidemiology Unit, Department of Public Health and Primary Care, University of Cambridge, Cambridge, UK
7. Victor Phillip Dahdaleh Heart and Lung Research Institute, University of Cambridge, Cambridge, UK
8. Health Data Research UK Cambridge, Wellcome Genome Campus and University of Cambridge, Cambridge, UK
9. British Heart Foundation Centre of Research Excellence, University of Cambridge, Cambridge, UK
10. National Institute for Health Research Blood and Transplant Research Unit in Donor Health and Genomics, University of Cambridge, Cambridge, UK
11. NHS Blood and Transplant–Oxford Centre, John Radcliffe Hospital and Radcliffe Department of Medicine, University of Oxford, John Radcliffe Hospital, Oxford, UK
12. Department of Bioinformatics, National Center for Global Health and Medicine, Tokyo, Japan

* Correspondence: R.X. (ruidong.xiang@agriculture.vic.gov.au) and M.I. (mi336@cam.ac.uk)

This file contains:
Supplementary Figures 1-13

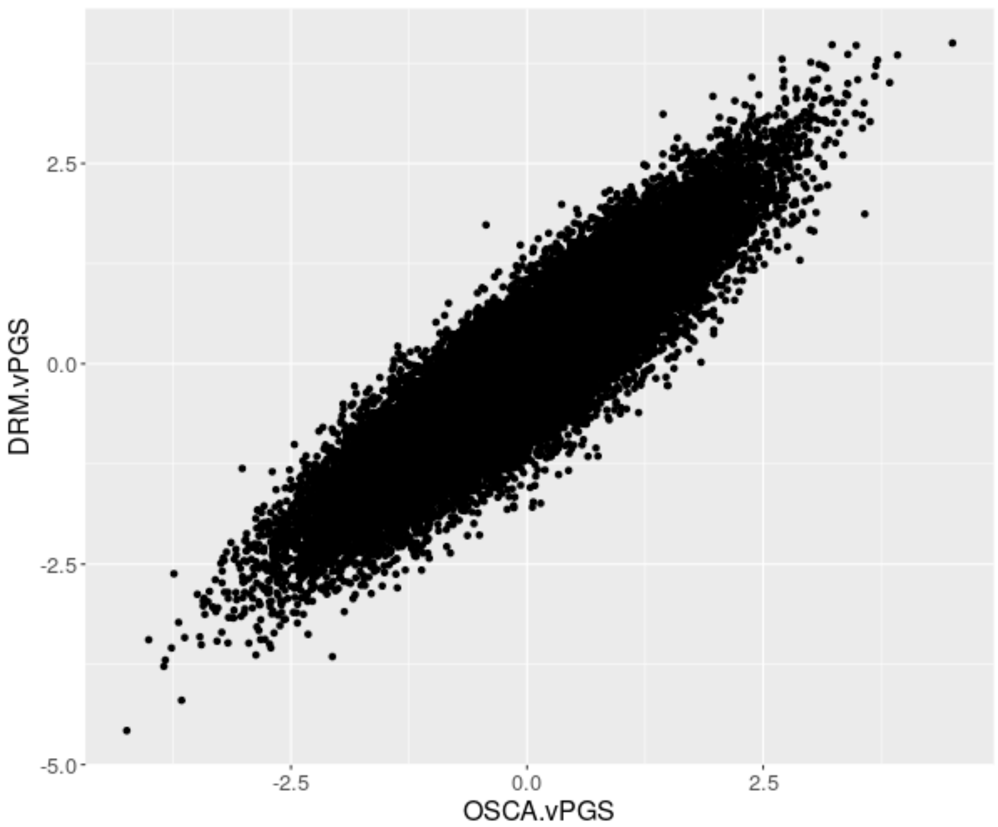

Supplementary Figure 1. Comparing vPGS computed using OSCA and DRM of mcv (mean corpuscular volume) in INTERVAL cohort. The Pearson correlation is 0.904.

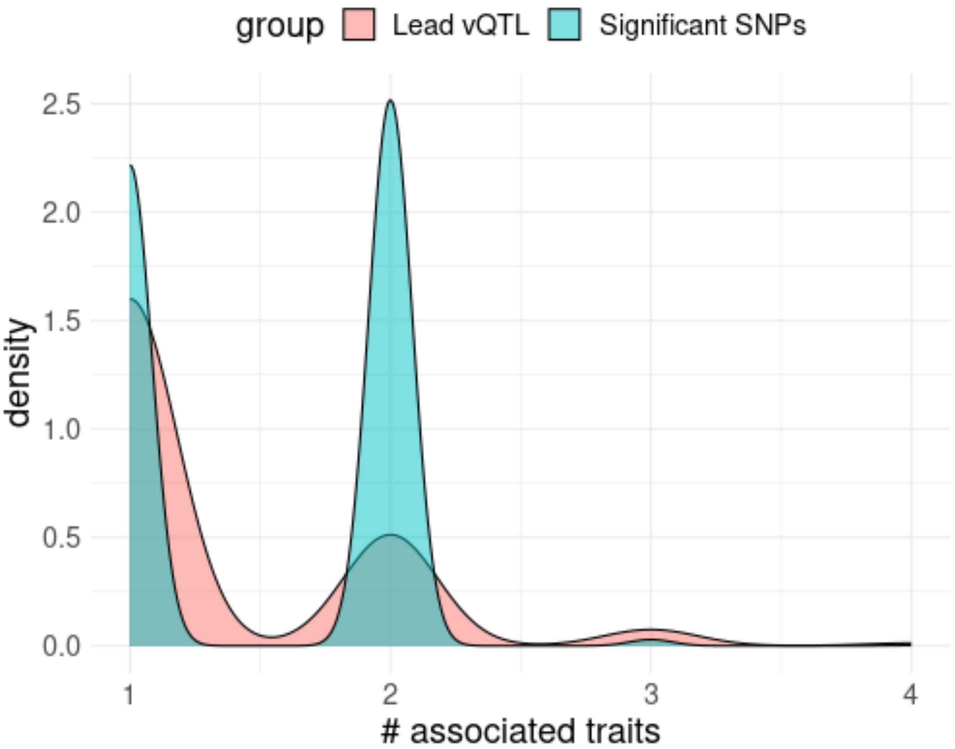

Supplementary Figure 2. Pleiotropy of vQTL. Lead QTLs were clumped with LD-$r^2 < 0.01$. Significant SNPs (not clumped) were associated with the variance of any traits with $p < 4.6 \times 10^{-9}$.

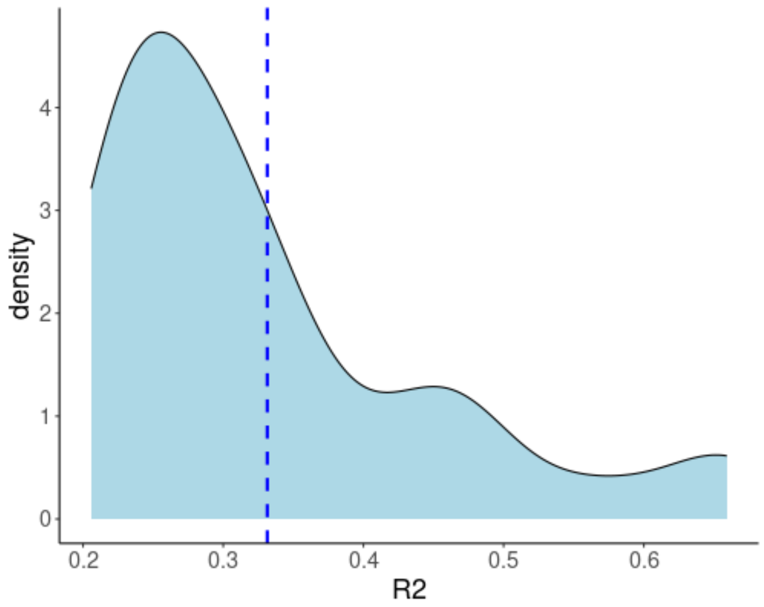

Supplementary Figure 3. Distribution of LD-r2 between novel lead vQTL identified in the current study and lead SNPs reported by Vuckovic et al 2020[1].

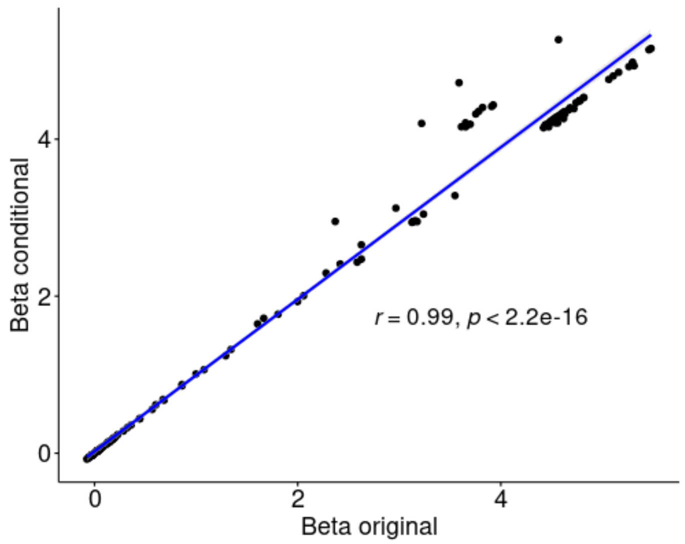

Supplementary Figure 4. Correlation of effects of 176 vQTL between original analyses and conditional analysis on the trait level.

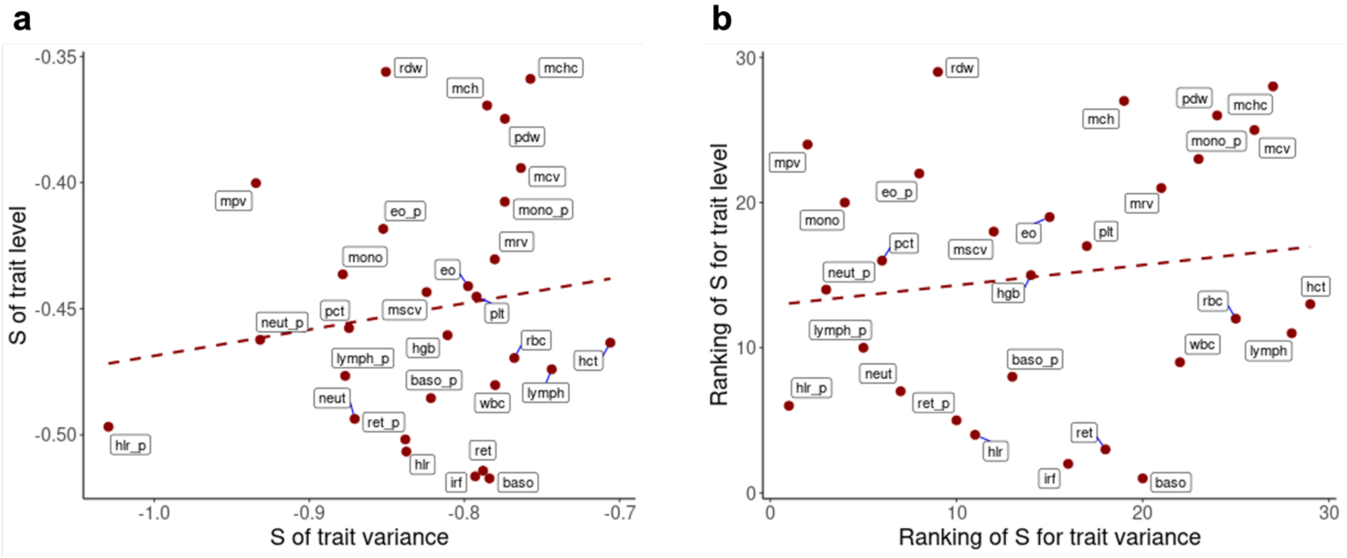

Supplementary Figure 5. Correlation between selection coefficient (S) between trait variance and level. **a**: Pearson correlation (*r* = 0.144, p = 0.46). **b**: correlation of ranking of S (*r* = 0.139, p = 0.47).

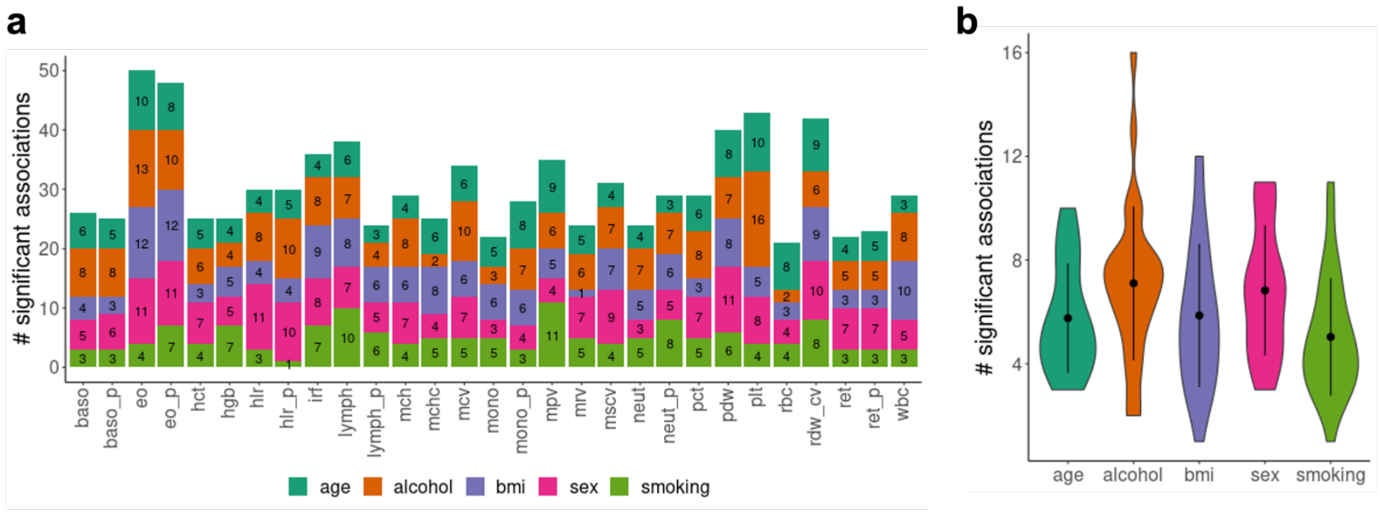

Supplementary Figure 6. Targeted GxE analysis using 176 lead vQTLs. **a**: bar plots counting the number of significant effects of interaction between lead vQTLs and 5 environmental factors. **b**: Distribution plot of the number of significant effects of interaction between lead vQTLs and 5 environmental factors across all blood cell traits. The violin plots' dot and error bars indicate the mean and standard deviation.

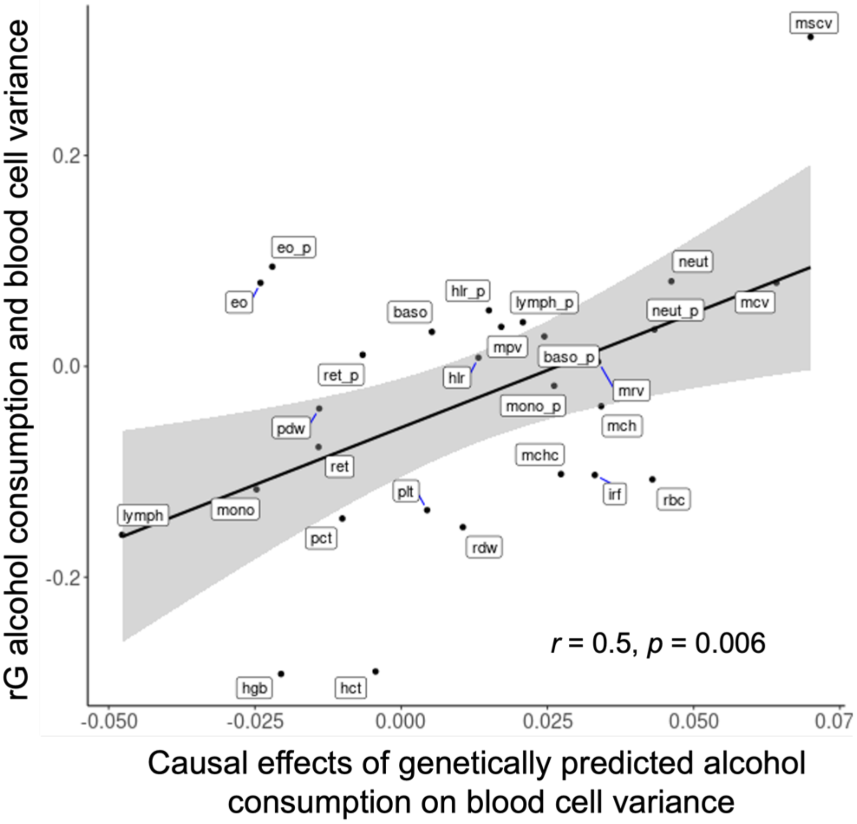

Supplementary Figure 7. Comparing genetic correlations between alcohol consumption and the variance of 29 blood cell traits (Y-axis) with Mendelian randomisation between alcohol consumption and the variance of 29 blood cell traits (X-axis). Genetic correlation was estimated using LD score regression and Mendelian randomisation was estimated using GSMR.

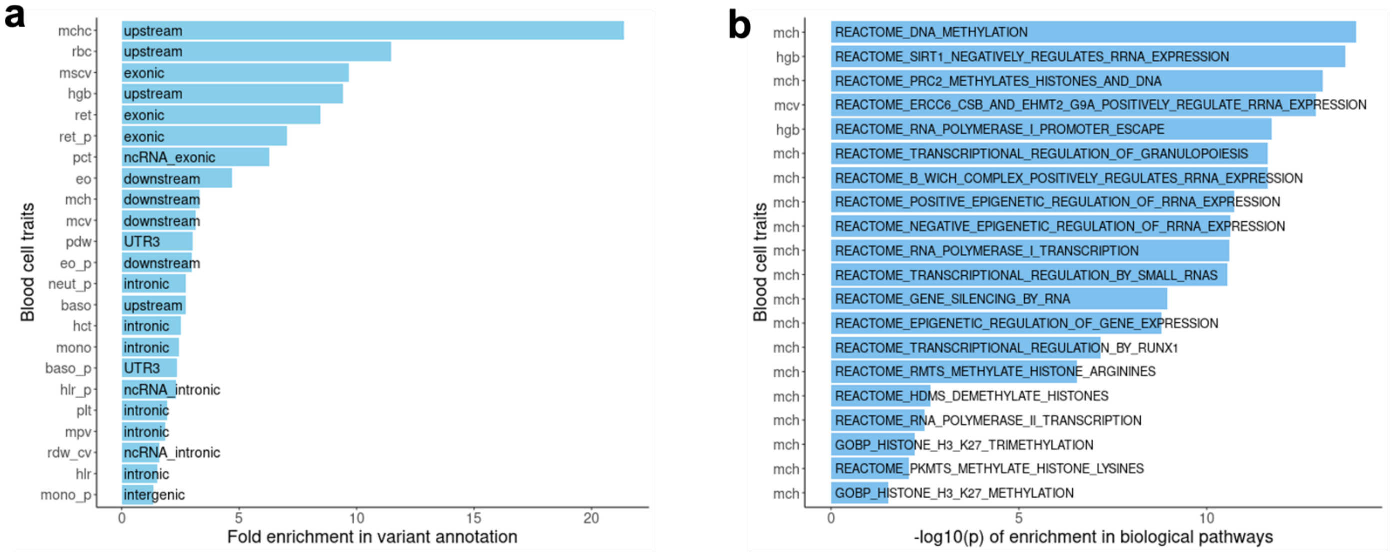

Supplementary Figure 8. a: Best enrichment of class of functional consequences (ANNOVAR) in lead vQTLs for each blood cell trait. **b**: Best enrichment of biological pathways related to gene regulation in lead vQTLs for each blood cell trait. All enrichment analyses were conducted using FUMA and only enrichments with multi-testing adjusted p-value < 0.05 are shown.

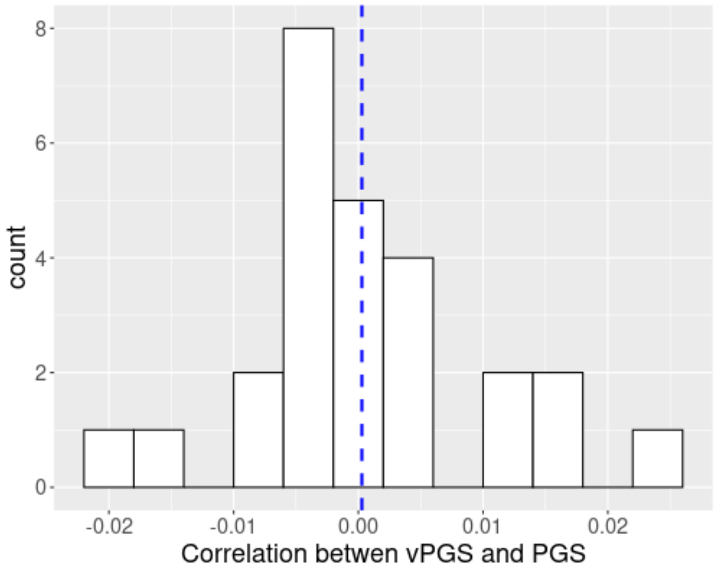

Supplementary Figure 9. Distribution of correlation between vPGS and PGS across 27 traits in INTERVAL. The blue line indicates the mean (*r* = 0.00028) of correlation across traits.

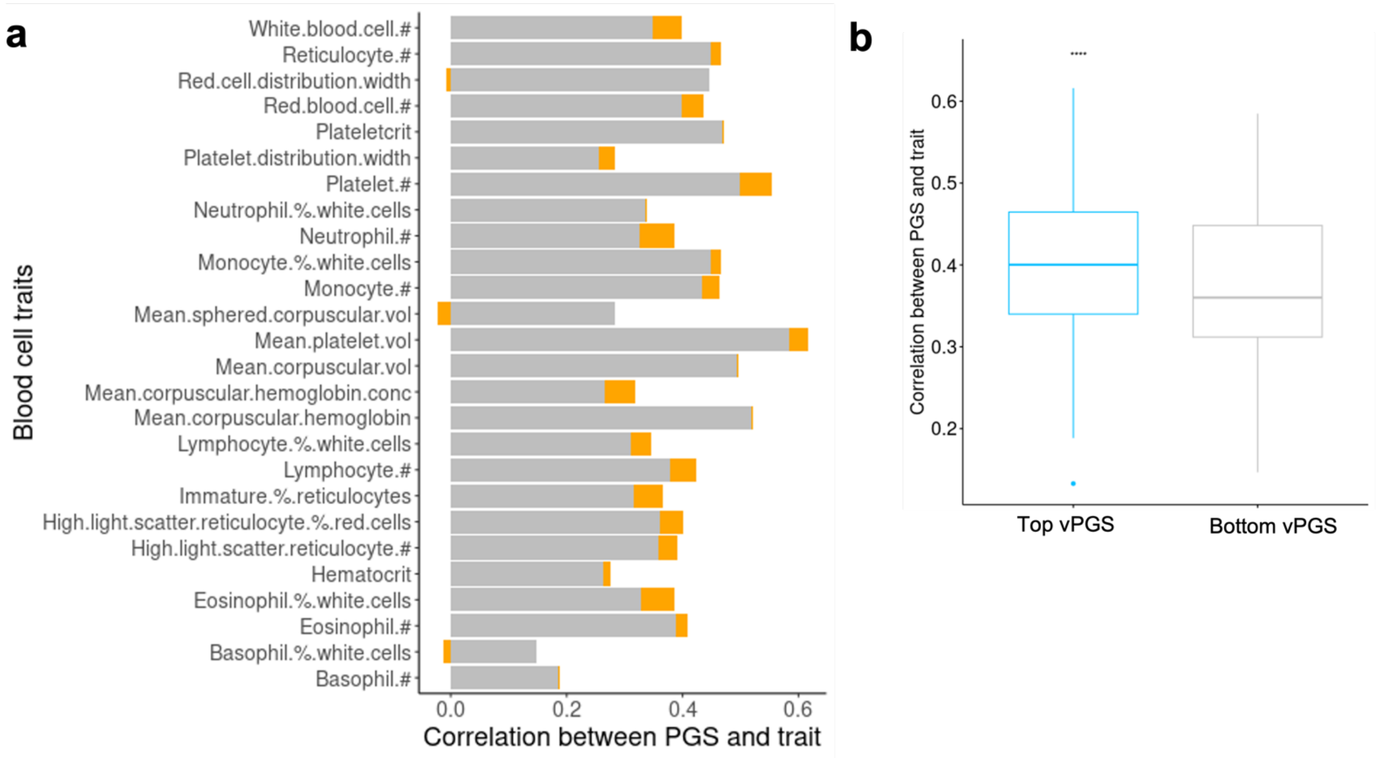

Supplementary Figure 10. The variation in the accuracy of PGSs for 27 blood cell traits (spearman correlation) between the top and bottom vPGS groups. **a**: Accuracy of PGS in the top vPGS group (more variable group, grey colour) and the difference (orange) of PGS between the top vPGS group and the bottom vPGS group (less variable group). #: count; % percentage; vol: volume; conc: concentration. **b**: difference of accuracy of PGS between the top and bottom vPGS groups across 27 blood cell traits. ****: p (2-sided test) < 0.0001. Note that panel **b** is a summary of individual trait data from panel **a**. For each box, the minimum is the lowest point, the maximum is the highest point, whiskers are maxima 1.5 times of interquartile range, the bottom bound, middle line and top bound of the box are the 25th percentile, median and the 75th percentile, respectively.

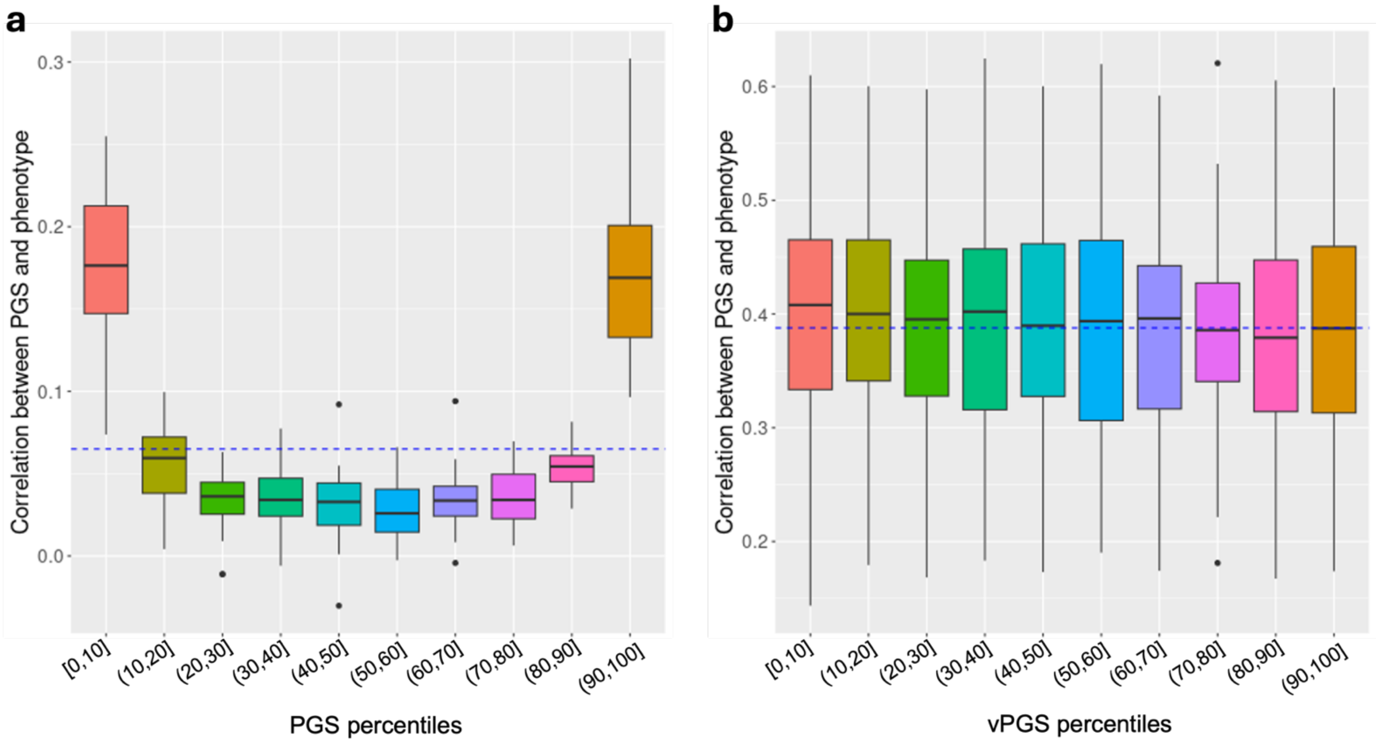

Supplementary Figure 11. Comparing the accuracy of PGS of blood cell traits across PGS bins (**a**) and vPGS bins (**b**). Accuracy is the Pearson correlation between PGS and blood cell trait. The INTERVAL cohort (N=40,466) was stratified by PGS bins (**a**) and vPGS bins (**b**) with percentile (0,10] being the highest PGS value (i.e. most variable vPGS) and percentile (90,100] being the lowest. In each bin, the box represents the distribution of the PGS prediction accuracy across 26 blood cell traits. Blue dashed lines represent the averaged PGS accuracy between 10 bins of PGS (**a**) and vPGS (**b**). For each box, the minimum is the lowest point, the maximum is the highest point, whiskers are maxima 1.5 times of interquartile range, the bottom bound, middle line and top bound of the box are the 25th percentile, median and the 75th percentile, respectively.

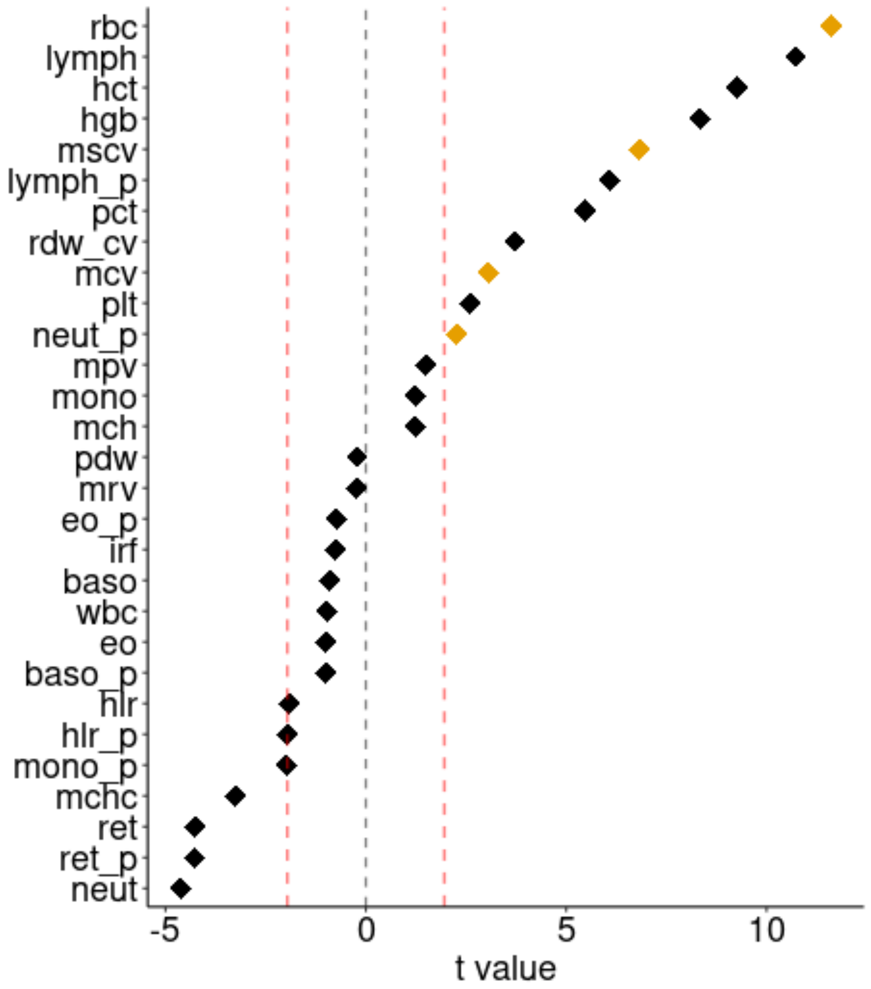

Supplementary Figure 12. Associating phenotypic variances in 29 blood cell phenotypes with alcohol consumption using Levene's test in the UK Biobank. t value = effects (of alcohol consumption) / se. Orange diamonds indicate these traits had $p < 0.05$ and the same effect direction with results from Mendelian randomisation (GSMR) between alcohol consumption (exposure) and blood cell traits (outcome). The red dashed line indicates values of -1.96 and +1.96, which equals p (2-sided) < 0.05 for the association test.

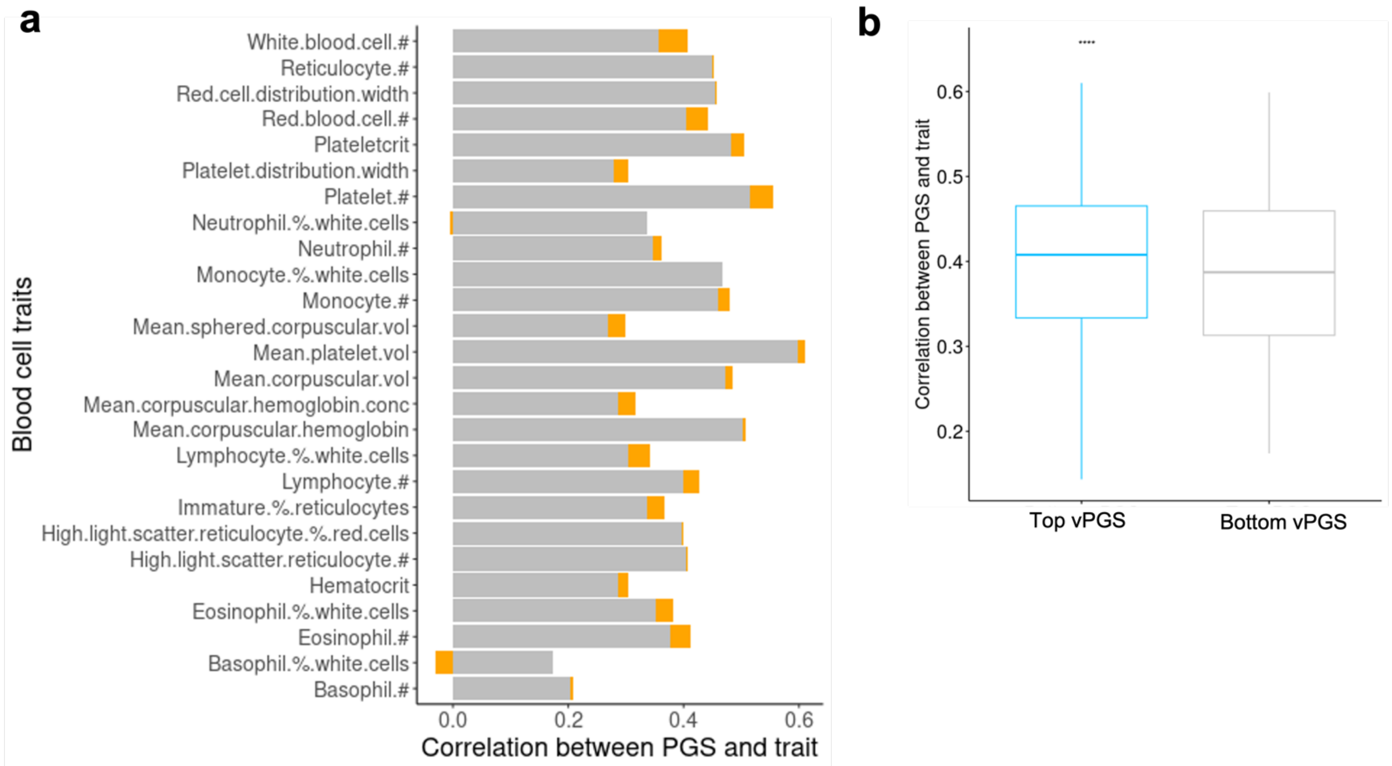

Supplementary Figure 13. The variation in the accuracy of PGSs for 27 blood cell traits (Pearson correlation) between the top 10% and bottom 10% vPGS group. **a**: Accuracy of PGS in the top vPGS group (more variable group, grey colour) and the difference (orange) of PGS between the top vPGS group and the bottom vPGS group (less variable group). #: count; % percentage; vol: volume; conc: concentration. **b**: Difference of accuracy of PGS between the top and bottom vPGS groups across 27 blood cell traits. ****: p (2-sided test) < 0.0001. Note that panel **b** is a summary of individual trait data from panel **a**. For each box, the minimum is the lowest point, the maximum is the highest point, whiskers are maxima 1.5 times of interquartile range, the bottom bound, middle line and top bound of the box are the 25th percentile, median and the 75th percentile, respectively.

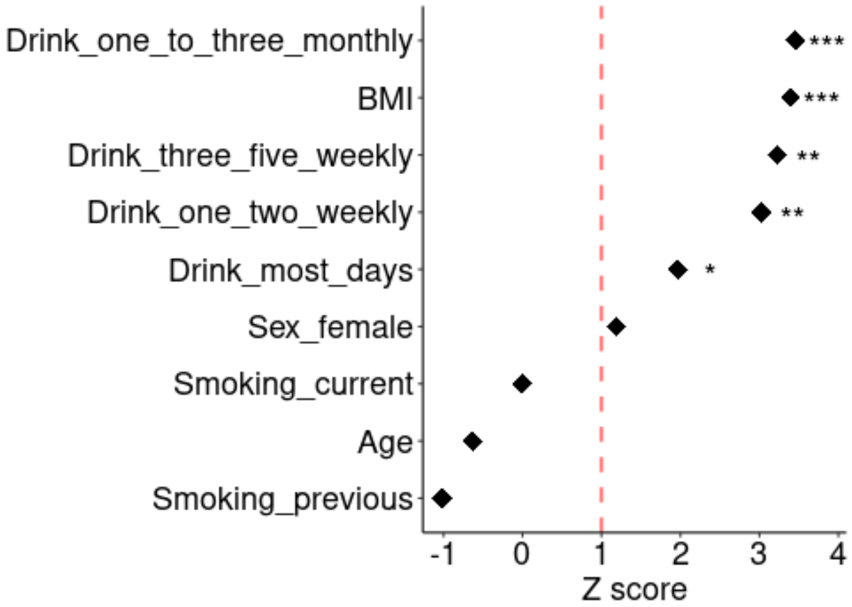

Supplementary Figure 14. Association between BMI, age, alcohol intake and smoking and individuals to be genetically variable across 27 blood cell traits in INTERVAL. Z score = beta (effects) / se (standard deviation). * (nominal Z-test signifiance): $p < 0.05$; **: $p < 0.01$; ***: $p < 0.001$ and **** $p < 0.0001$.

References:

1 Vuckovic, D. *et al.* The polygenic and monogenic basis of blood traits and diseases. *Cell* **182**, 1214-1231. e1211 (2020).
