# Supplementary material for: Genome-wide analyses of variance in blood cell phenotypes provide new insights into complex trait biology and prediction
Source: Nat Commun. 2025 May 7;16:4260. doi: 10.1038/s41467-025-59525-4 (PMC12059119; doi:10.1038/s41467-025-59525-4)
Supplement: Supplementary file 2 — Reporting Summary [file 41467_2025_59525_MOESM2_ESM.pdf]

nature portfolio

Corresponding author(s): Michael Inouye

Last updated by author(s): Mar 28, 2025

Reporting Summary

Nature Portfolio wishes to improve the reproducibility of the work that we publish. This form provides structure for consistency and transparency in reporting. For further information on Nature Portfolio policies, see our Editorial Policies and the Editorial Policy Checklist.

Statistics

For all statistical analyses, confirm that the following items are present in the figure legend, table legend, main text, or Methods section.

n/a	Confirmed	
☐	☒	The exact sample size (*n*) for each experimental group/condition, given as a discrete number and unit of measurement
☐	☒	A statement on whether measurements were taken from distinct samples or whether the same sample was measured repeatedly
☐	☒	The statistical test(s) used AND whether they are one- or two-sided<br>*Only common tests should be described solely by name; describe more complex techniques in the Methods section.*
☐	☒	A description of all covariates tested
☐	☒	A description of any assumptions or corrections, such as tests of normality and adjustment for multiple comparisons
☐	☒	A full description of the statistical parameters including central tendency (e.g. means) or other basic estimates (e.g. regression coefficient) AND variation (e.g. standard deviation) or associated estimates of uncertainty (e.g. confidence intervals)
☐	☒	For null hypothesis testing, the test statistic (e.g. *F*, *t*, *r*) with confidence intervals, effect sizes, degrees of freedom and *P* value noted<br>*Give P values as exact values whenever suitable.*
☐	☒	For Bayesian analysis, information on the choice of priors and Markov chain Monte Carlo settings
☒	☐	For hierarchical and complex designs, identification of the appropriate level for tests and full reporting of outcomes
☐	☒	Estimates of effect sizes (e.g. Cohen's *d*, Pearson's *r*), indicating how they were calculated

Our web collection on statistics for biologists contains articles on many of the points above.

Software and code

Policy information about availability of computer code

Data collection	No specific software was used to collect the data.
Data analysis	Code using existing software is accessible via https://github.com/rxiangr/vQTL (Zenodo: https://sandbox.zenodo.org/account/settings/github/repository/rxiangr/vQTL; DOI: 10.5072/zenodo.187912). vQTL mapping used OSCA (v0.46): https://yanglab.westlake.edu.cn/software/osca/#Overview; genetic correlation analysis used LDSC (v1.01): https://github.com/bulik/ldsc; pleiotropy analysis: https://github.com/rondolab/HOPS. Mendelian randomisation used GSMR (v1.1.1): https://yanglab.westlake.edu.cn/software/gsmr/, MR-PRESSO: https://github.com/rondolab/MR-PRESSO and MendelianRandomisation (v0.1): https://cran.r-project.org/web/packages/MendelianRandomization/index.html; Analysis of selection used GCTB-BayesS (v2.05): https://cnsgenomics.com/software/gctb/#SummaryBayesianAlphabet; vPGS analysis used PRSICE-2 (v2.3.5): https://choishingwan.github.io/PRSice/ and plink2 (alpha4): https://www.cog-genomics.org/plink/2.0/; multi-trait GBLUP used SMTpred: https://github.com/uqrmaie1/smtpred; significance tests of R2 increase used r2redux (v1.0.18): https://github.com/mommy003/r2redux; logistic regression analysis used glm(): https://www.rdocumentation.org/packages/stats/versions/3.6.2/topics/glm.

For manuscripts utilizing custom algorithms or software that are central to the research but not yet described in published literature, software must be made available to editors and reviewers. We strongly encourage code deposition in a community repository (e.g. GitHub). See the Nature Portfolio guidelines for submitting code & software for further information.

Data

Policy information about availability of data

All manuscripts must include a data availability statement. This statement should provide the following information, where applicable:

- Accession codes, unique identifiers, or web links for publicly available datasets
- A description of any restrictions on data availability
- For clinical datasets or third party data, please ensure that the statement adheres to our policy

All data described are available through the UK Biobank subject to approval from the UK Biobank access committee. See https://www.ukbiobank.ac.uk/enable-your-research/apply-for-access for further details. INTERVAL study data from this paper are available to bona fide researchers from helpdesk@intervalstudy.org.uk and information, including the data access policy, is available at http://www.donorhealth-btru.nihr.ac.uk/project/bioresource.

Research involving human participants, their data, or biological material

Policy information about studies with human participants or human data. See also policy information about sex, gender (identity/presentation), and sexual orientation and race, ethnicity and racism.

Reporting on sex and gender	In the manuscript, we use the term sex to describe the biological attribute. Sex was reported at the time of enrolment to UK Biobank or INTERVAL. In the UKB cohort, there are 273353 females and 229106 males. We do not share individual-level data. Based on the reported sex, in the INTERVAL cohort, there are 22685 females and 22356 males. The all analyses were applied to all sexes. Sex was used as a covariate in the regression of vQTL mapping.
Reporting on race, ethnicity, or other socially relevant groupings	Only White British individuals are used in the study and 10 genetic PCs were used in the UK Biobank and INTERVAL to correct population structures.
Population characteristics	UK Biobank is a cohort of approximately 500,000 participants from the general UK population. Participants were between age 40 and 69 at recruitment (median 58 years of age; 54% women). Age, sex, principal components of genetic ancestry, body mass index, smoking status were used as covariates. INTERVAL is a cohort of approximately 50,000 participants nested within a randomised trial studying the safety of varying the frequency of blood donation (ISRCTN24760606). Participants were blood donors aged 18 years and older (median 44 years of age; 50% women).
Recruitment	UK Biobank participants were between age 40 and 69 at recruitment (median 58 years of age; 54% women) and accepted an invitation to attend one of the assessment centres that were established across the United Kingdom between 2006 and 2010. Interval participants were blood donors aged 18 years and older recruited between 2012 and 2014 from 25 NHS Blood and Transplant centers.
Ethics oversight	UK Biobank has approval from the North West Multi-centre Research Ethics Committee (MREC) as a Research Tissue Bank (RTB). Access to UK Biobank data was granted by UK Biobank under application ID 7439. INTERVAL was approved by the National Research Ethics Service (11/EE/0538).

Note that full information on the approval of the study protocol must also be provided in the manuscript.

Field-specific reporting

Please select the one below that is the best fit for your research. If you are not sure, read the appropriate sections before making your selection.

☒ Life sciences ☐ Behavioural & social sciences ☐ Ecological, evolutionary & environmental sciences

For a reference copy of the document with all sections, see nature.com/documents/nr-reporting-summary-flat.pdf

Life sciences study design

All studies must disclose on these points even when the disclosure is negative.

Sample size	Sample size was determined by the available data in the UK Biobank (N=408,111) and INTERVAL (N=40,466) cohorts, excluding measures that did not pass quality control. No sample size calculations were performed in this work, but such sample sizes have proven to be more than adequate in numerous previous studies using such cohorts.
Data exclusions	Only measures that did not pass quality control were excluded.
Replication	The PGSs and vPGSs of blood cell traits were trained in the UK Biobank data and then predicted into the INTERVAL cohort to test the prediction accuracy.
Randomization	Not applicable.
Blinding	Not applicable.

Reporting for specific materials, systems and methods

We require information from authors about some types of materials, experimental systems and methods used in many studies. Here, indicate whether each material, system or method listed is relevant to your study. If you are not sure if a list item applies to your research, read the appropriate section before selecting a response.

Materials & experimental systems

n/a	Involved in the study
☒	☐ Antibodies
☒	☐ Eukaryotic cell lines
☒	☐ Palaeontology and archaeology
☒	☐ Animals and other organisms
☐	☒ Clinical data
☒	☐ Dual use research of concern
☒	☐ Plants

Methods

n/a	Involved in the study
☒	☐ ChIP-seq
☒	☐ Flow cytometry
☒	☐ MRI-based neuroimaging

Clinical data

Policy information about clinical studies

All manuscripts should comply with the ICMJEguidelines for publication of clinical research and a completedCONSORT checklist must be included with all submissions.

Clinical trial registration	Access to UK Biobank data was granted by UK Biobank under application ID 7439. INTERVAL (NCT01610635).
Study protocol	UK Biobank: https://doi.org/10.1371/journal.pmed.1001779; INTERVAL: https://doi.org/10.1186/1745-6215-15-363
Data collection	In both UKB and INTERVAL, blood samples were predominantly collected and analyzed from the baseline visit. Anthropometric measurements and health and lifestyle questionnaires were also collected. In INTERVAL, participants were blood donors aged 18 years and recruited between 2012 and 2014 from 25 NHS Blood and Transplant centers. In UK Biobank, participants accepted an invitation to attend one of the assessment centers that were established across the United Kingdom between 2006 and 2010.
Outcomes	The outcome of this work is identified vQTLs associated with variance of blood cell traits and the knowledge of how they can be used to discover new biology and improve PGSs of blood cell traits.

Plants

Seed stocks	Not applicable.
Novel plant genotypes	Not applicable.
Authentication	Not applicable.
