# Supplementary material for: Genome-wide analyses of variance in blood cell phenotypes provide new insights into complex trait biology and prediction
Source: Nat Commun. 2025 May 7;16:4260. doi: 10.1038/s41467-025-59525-4 (PMC12059119; doi:10.1038/s41467-025-59525-4)
Supplement: Supplementary file 3 — Description of Additional Supplementary Files [file 41467_2025_59525_MOESM3_ESM.pdf]

<u>Description of Additional Supplementary Files</u>

Supplementary Data 1. Summary of blood cell traits in the UK Biobank and INTERVAL.

Supplementary Data 2. LDSC estimated genome-wide inflation factors for vQTL mapping of blood cell traits.

Supplementary Data 3. Lead vQTLs for each blood cell trait.

Supplementary Data 4. Count of number of significant vQTLs ($p < 4.6x10-9$ from vQTL GWAS based on Levene's test) for each trait.

Supplementary Data 5. Results of pleiotropic analysis of vQTLs using HPOS.

Supplementary Data 6. Genetic correlation (rg based on LDSC) between the level and variance for each blood cell trait. P: nominal p value; padj: multi-testing adjust p-value.

Supplementary Data 7. BayesS estimated selection coefficient (S) for blood cell trait variance and level. P-values are 2-sided and based on the method of GCTB (https://cnsgenomics.com/software/gctb/#Overview).

Supplementary Data 8. FUMA annotation of vQTLs for each blood cell trait.

Supplementary Data 9. FUMA enrichment analysis of vQTLs for each blood cell trait. P: nominal p-values from the Fisher exact test implemented in FUMA (https://fuma.ctglab.nl/). adjP: multi-testing adjusted p-values.

Supplementary Data 10. False discovery rate for targeted GxE analysis using lead vQTLs and environmental factors. P_threshold: different p-value (2-sided) threshold to call significant GxE associations. Only vQTLs with MAF >= 0.0001 were considered in the analysis.

Supplementary Data 11. Estimates of Mendelian Randomisation between alcohol consumption (exposure) and blood cell trait variance (outcome) and the genetic correlation between alcohol consumption and blood cell trait variance.

Supplementary Data 12. full results of analysing the interaction between PGS and vPGS. For each trait, PGS is the main effect of conventional polygenic score, vPGS is the main effect of variance polygenic score and PGS:vPGS is the interaction. p: significance of the effects (2-side test).

Supplementary Data 13. Normality test for each blood cell traits using Kolmogorov-Smirnov Test. ks.D is the test statistic and the p value (1-sided) >=0.05 is interpreted as the data is likely from a normal distribution.
